# Effect of filtration on elimination of turbidity and changes in volatile compounds concentrations in plum distillates

**DOI:** 10.1007/s13197-019-03682-0

**Published:** 2019-03-06

**Authors:** Maria Balcerek, Katarzyna Pielech-Przybylska, Urszula Dziekońska-Kubczak, Piotr Patelski, Mateusz Różański

**Affiliations:** Department of Spirit and Yeast Technology, Institute of Fermentation Technology and Microbiology, Lodz University of Technology, Wolczanska 171/173, 90-924 Lodz, Poland

**Keywords:** Fruit distillate, Turbidity, Volatile compounds, Fatty acid esters, Cold storage

## Abstract

The purpose of this study was to investigate the effect of alcoholic strength by volume (ASV) and storage conditions on turbidity in plum brandies. Different types of filter sheet were also tested for their effects on turbidity, as well as on the chemical composition and organoleptic characteristics of the distillates. The raw materials used were two plum distillates with initial ASVs of 76.77% v/v and 81.92% v/v. The distillates were diluted to alcohol contents of 37.5%, 40% and 50% v/v and stored under various conditions for 64 days. Filtration was performed using two depth filter sheets, with nominal retention rates of 0.40–0.48 μm and 0.80 μm, or with an activated carbon-based filter sheet. The lowest turbidity was observed in samples stored at ambient temperature with an ASV of 50% v/v. Reducing the alcohol content and storage temperature caused turbidity to increase. Samples prepared from distillate with an initial alcohol content of 76.77% v/v were characterized by significantly higher turbidity than those produced from spirit with an initial ASV of 81.92% v/v. Lowering the storage temperature resulted in a larger decrease in the concentration of volatile compounds after filtration. Use of an activated carbon filter sheet caused the greatest decrease in the majority of volatiles. Use of a filter sheet with a nominal retention rate of 0.80 μm led to the greatest improvement in the organoleptics of the tested plum distillates.

## Introduction

Fruit distillates produced by alcoholic fermentation of fleshy fruits or their musts are of interest as spirit beverages, due to their rich flavours and aromas. Generally, such beverages are made from raw spirits, which are not purified by rectification but aged in wooden barrels, where their qualities are allowed to develop naturally through certain chemical reactions (Regulation (EC) no. 110/2008). The volatile compounds in the spirits are what give the end product its specific character. However, when the alcohol content is below 45% v/v, they can also cause undesirable turbidity, commonly referred to as haze or cloudiness, during storage (especially under refrigerated conditions) (Miljić et al. 2013). Turbidity is associated with a decrease in the solubility of volatile compounds, such as higher alcohols, fatty acid esters (ethyl laurate, ethyl palmitate, ethyl palmitoleate, ethyl myristate) and others, especially at lower temperatures (Hsieh et al. 2014; Pirie et al. 2000). The fatty acid esters behave as surfactants, because, as well as a hydrophilic group, they have long hydrophobic carbon chains that prevent them from mixing with water. Under non-mixing conditions, these fatty esters behave as micelles—spherical clumps of lipid molecules in which the hydrophobic carbon tail points toward the center, away from the water. Given the role that fatty acid esters play in causing cloudiness, it is important to consider ways in which their quantities can be reduced (Carrillo and Cristobal 2015).

As well as volatile compounds, metal ions such as Cu, Fe, Na, Mg, K and others may also cause spirit hazing (Adam et al. 2002; Fernandes et al. 2013; Ibanez et al. 2008). Metal ions in white vodkas originate primarily from water (Krosnijs and Kuka 2003), distillation apparatuses, storage tanks and glass bottles. In the case of flavoured spirit beverages, sources of metal ions include plant raw materials-based macerates, fruit musts and other flavoring agents (Mayer et al. 2003). Spirits such as cognac or whisky may contain metal ions (such as Cu), imparted by the copper stills (alembics) used for distillation (Adam et al. 2002).

There are several ways to reduce the quantities of compounds responsible for cloudiness in raw distillates and spirit beverages. One solution uses activated carbon (Mukhin et al. 2009; Ng et al. 1996), a strongly adsorbent and hydrophobic material (Ligor et al. 1998). Thanks to these properties, activated carbon is able to trap volatile compounds (Balcerek et al. 2017a, b) and absorb organic compounds found in alcoholic beverages such as spirits. It also catalyzes a number of chemical reactions (oxidation, isomerization, esterification, etc.). The simplest procedure is to mix activated carbon with the spirit solution and separate the adsorbent by filtration. Another possibility is to pass the ethanol solution through a large amount of activated carbon/charcoal in a packed column. The spirit should flow through the columns at such a speed that ensures sufficient contact between the solid and the liquid phase (Perederii et al. 2011; Siristova et al. 2013).

Cold filtration is another process that may effectively eliminate problems connected with turbidity in spirit beverages. The distillate is diluted to the desired alcohol strength and frozen at a temperature of between − 5 and − 7 °C for a few days. It is then filtered using cellulose filters, carbon filters, diatomaceous earth filters or candle filters (Brüning 2018; Glaub et al. 1998; Hsieh et al. 2014; Pirie et al. 2000). The filtration materials should be chosen carefully, to prevent the loss of flavour compounds which give the spirit its characteristic qualities.

The aim of the present study was to evaluate the effect of ethanol concentration, temperature and the presence or absence of daylight during storage on turbidity in plum distillates with different initial ASVs. Different types of filtration sheet were also assessed in terms of their ability to reduce the concentration of volatile compounds in the plum distillates, eliminate turbidity and improve the organoleptic characteristics of the spirit beverages.

## Materials and methods

### Materials

The raw materials used were plum distillates produced on an industrial scale by the Polish Company + H2O, Chociszew near Szadek, Poland, with initial ASVs of 76.77% v/v and 81.92% v/v. The samples were treated using three types of commercial filtration sheet (Eaton, USA):

- EUROPOR^®^ K40 (nominal retention rate from 0.40 to 0.48 μm)—this depth filter sheet type is particularly suitable for cold sterile bottling or storage of beverages. Excellent retention capacity for colloidal substances is ensured by the fine pore structure combined with adsorptive electrokinetic properties of EUROPOR^®^ K depth filter sheets (http://bevitas.de/files/TI_EUROPOR-Range_en.pdf) (Begerow^®^ Product Line 2018).
- BECO^®^ SELECT A20 (nominal retention rate 0.80 µm)—this filter sheet provides excellent filtration of spirits based on pomaceous fruit, stone fruit, soft fruit, yeast, grapes, juniper berries (Gin, Genever) and grain (Vodka, Aquavit, Steinhäger). It reliably retains very fine particles and higher fatty acids, making the BECO^®^ SELECT A20 filter sheet particularly suitable for haze-free storage and filling (http://www.eaton.com) (Filtration Products-Eaton 2018).
- BECO^®^ ACF 07—the activated carbon in this depth filter sheet is a micro-porous inert material, which is acid-washed and steam-activated. When products are cleaned or decolorized, a physical bond is created between the interior surfaces of the activated carbon and impurities or colored substances. Since this bond is largely non-polar, the BECO^®^ ACF 07 filter sheet has a great affinity to organic molecules (http://www.eaton.com) (Filtration Products-Eaton 2018).

### Preparation of samples

The plum distillates were diluted with deionized water (Simplicity^®^ Ultrapure Water System, 18.2 MΩ∙cm resistivity (25 °C) at 0.5 L/min, Merck Millipore) from initial ASVs of 76.77% v/v and 81.92% v/v to 37.5, 40 and 50% v/v. Next, the samples were placed in glass bottles and stored under the following conditions, for 64 days:

- ambient temperature (+ 20 ± 2 °C), daylight,
- ambient temperature (+ 20 ± 2 °C), darkness,
- cold storage (+ 8 °C), darkness,
- temperature close to freezing (− 16 °C for ASVs of 37.5% v/v and 40% v/v, − 25 °C for ASV of 50% v/v), darkness.

After 10, 20, 30, 34, 42, 50, 57 and 64 days the turbidity of the samples was measured. After 64 days, the samples were filtered through filter sheets and then subjected to turbidity measurements, as well as to chromatographic and organoleptic analysis. The control samples were the distillates before treatment. Prior to filtration, the used filter sheets were rinsed with deionized water for 10 min in the direction of flow.

### Analytical methods

Turbidity was determined according to ISO 7027 (2016) using a Hach 2100p turbidimeter. The level of turbidity was expressed in nephelometric turbidity units (NTU), with the use of formazin suspensions in concentrations of 0.4, 0.8, 1,2, 1.6 and 2 NTU as a standard. Chromatographic analysis of the volatile compounds in the distillates was performed using a GC apparatus (Agilent 7890A, USA) with a mass spectrometer (Agilent MSD 5975C, USA), following the method described by Pielech-Przybylska et al. (2016).

### Sensory evaluation

Before and after storage and filtration through different filter sheets, the plum distillate samples were evaluated organoleptically by panel of 12 semi-trained judges using a nine point hedonic scale (Peryam and Pilgrim 1957). The panel was composed of employees at the Institute of Fermentation Technology and Microbiology of Lodz University of Technology. The assessors (5 females, 7 males) were aged 35–60. The panelists rated 4 sensorial qualities (color, clearness, odor, taste) as well as overall acceptability. Before sensory evaluation, all the tested samples were diluted with deionized water to ASV of 37.5% v/v, to eliminate differences in alcohol content.

### Statistical analysis

All samples were prepared and analyzed in triplicate. The results were expressed as average ± SD. Statistical analyses were performed using STATISTICA 10 software (Tibco Software, Palo Alto, CA, USA). The results were evaluated using analysis of variance (ANOVA), followed by Tukey’s post hoc test to verify statistical differences with a significance level of *p* = 0.05.

## Results and discussion

### Chemical composition of tested plum distillates

The plum distillates were produced by a Polish manufacturer of plum brandies on an industrial scale. The distillates were obtained using a one-column continuous apparatus and showed initial ASVs of 76.77% v/v and 81.92% v/v, respectively. These ASVs met the requirements for concentrations of ethanol in fruit spirits, as set out in Regulation (EC) no. 110 of the European Parliament and the Council (2008), which states that the ASV of fruit spirits should be less than 86% v/v.

The compositions of the volatile compounds in the obtained plum distillates (Table 1) differed significantly and were closely related to their ASVs. The distillate with an ASV of 81.92% v/v was characterized by lower concentrations of the majority of volatile compounds than the sample with an ASV of 76.77% v/v. The exceptions were in the concentrations of higher alcohols—for instance, there was no difference between the two distillates in terms of their contents of 2-methyl-1-butanol or 3-methyl-butanol (*p* > 0.05). The concentrations of other alcohols in the distillate with the higher ASV (81.92% v/v) were approx. 24% (1-propanol, 2-methyl-1-propanol) to 60% (2-phenylethanol) lower in comparison to the spirit with an alcohol content of 76.77% v/v. This was probably due to the fact that compounds with high molecular weights have a tendency to distil in the tail fractions (Matias-Guiu et al. 2016). This had been confirmed by the results of a previous study by the authors, in which the highest concentration of 2-phenylethanol was found in plum distillate with a 70% v/v alcohol content, and the lowest in a fraction with an ASV of 90% v/v (Balcerek et al. 2017a, b).

**Table 1:** Chemical composition of tested plum distillates

Compound [mg/L alcohol 100% v/v]	Distillate with initial ASV of 76.77% v/v	Distillate with initial ASV of 81.92% v/v
Alcohols		
1-propanol	515.30^b^ ± 43.20	395.52^a^ ± 29.50
2-methyl-1-propanol	95.22^b^ ± 8.92	70.89^a^ ± 9.30
1-butanol	6.90^b^ ± 0.54	1.65^a^ ± 0.23
2-methyl-1-butanol	704.15^a^ ± 24.59	696.40^a^ ± 36.05
3-methyl-1-butanol	2977.86^a^ ± 134.8	2858.05^a^ ± 160.29
2-phenylethanol	2.00^b^ ± 0.18	0.80^a^ ± 0.19
Methanol	49.59^b^ ± 6.74	23.08^a^ ± 2.87
Esters		
Ethyl acetate	193.25^a^ ± 5.98	208.86^a^ ± 11.97
2-methylbutyl acetate	3.36^b^ ± 0.12	1.93^a^ ± 0.06
Isoamyl acetate	7.58^b^ ± 0.91	5.26^a^ ± 0.84
2-phenylethyl acetate	33.15^b^ ± 4.26	20.72^a^ ± 2.15
Ethyl propanoate	0.78^b^ ± 0.09	0.56^a^ ± 0.06
Ethyl butanoate	2.89^b^ ± 0.33	1.72^a^ ± 0.15
Ethyl 3-methylbutanoate	0.78^b^ ± 0.03	0.36^a^ ± 0.06
Ethyl benzoate	0.39^b^ ± 0.03	0.19^a^ ± 0.02
Ethyl hexanoate	2.85^a^ ± 0.30	2.51^a^ ± 0.38
Ethyl octanoate	10.29^b^ ± 0.18	8.47^a^ ± 0.95
2-methylpropyl octanoate	1.40^b^ ± 0.15	0.78^a^ ± 0.02
3-methylbutyl octanoate	1.60^b^ ± 0.13	1.23^a^ ± 0.09
Octyl octanoate	0.53^b^ ± 0.07	0.36^a^ ± 0.06
Decyl octanoate	0.22^a^ ± 0.04	0.18^a^ ± 0.02
Ethyl nonanoate	0.45^b^ ± 0.02	0.36^a^ ± 0.05
Ethyl decanoate	0.75^b^ ± 0.01	0.36^a^ ± 0.01
Isobutyl decanoate	0.58^b^ ± 0.03	0.32^a^ ± 0.08
Ethyl tetradecanoate	1.17^b^ ± 0.41	0.13^a^ ± 0.05
Carbonyl compounds and acetals		
Acetaldehyde	91.99^a^ ± 7.44	97.28^a^ ± 7.04
Isobutyraldehyde	2.24^a^ ± 0.69	4.03^b^ ± 0.97
Isovaleraldehyde	0.52^a^ ± 0.18	0.53^a^ ± 0.07
2-methylbutyraldehyde	0.68^a^ ± 0.15	0.78^a^ ± 0.12
Benzaldehyde	0.83^b^ ± 0.17	0.55^a^ ± 0.09
2,3-butanodione	0.52^a^ ± 0.43	1.53^b^ ± 0.67
Acetaldehyde diethyl acetal	155.81^a^ ± 42.89	145.78^a^ ± 65.68

Mean values in rows with different superscript lowercase letters are significantly different (*p* < 0.05), as analyzed by the Tukey’s post hoc test

Statistically significant (*p* < 0.05) differences in the concentrations of the majority of esters were also observed, depending on the alcohol contents of the tested distillates. The distillate with a higher ASV contained lower levels of these compounds, except for ethyl acetate and ethyl hexanoate.

The concentrations of carbonyl compounds in the distillates did not differ significantly (*p* > 0.05). This may be connected with their relatively high volatility, which means that they are present at higher concentrations in the initial fractions of distillates (Balcerek et al. 2017a, b). The only exceptions were benzaldehyde, 2,3-butanodione and isobutyraldehyde. The content of benzaldehyde was inversely correlated to the ethanol concentration; a higher ASV caused the content of benzaldehyde to decrease (*p* < 0.05). The concentrations of isobutyraldehyde and 2,3-butanodione were much stronger in the distillate with a higher ASV.

All of the tested plum distillates satisfied the requirements of EU Regulation (EC) no. 110/2008, which states that the quantity of volatile aroma compounds should be not less than 200 grams per hectoliter of 100% v/v alcohol. The concentrations of undesirable compounds, such as methanol, did not exceed the levels specified in the same regulation for plum distillates (*Prunus domestica* L.), at less than 1200 grams per hectoliter of 100% v/v alcohol.

### Turbidity changes in stored plum distillate samples

**Fig. 1:**
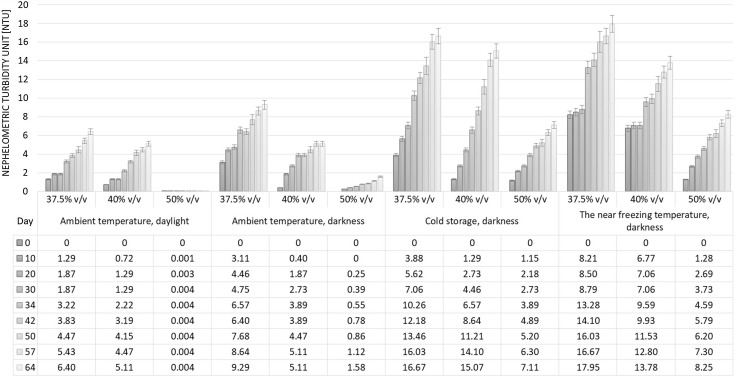
Effect of alcoholic strength by volume (ASV) and storage conditions on turbidity changes in solutions prepared from plum distillate with initial ASV of 76.77% v/v

**Fig. 2:**
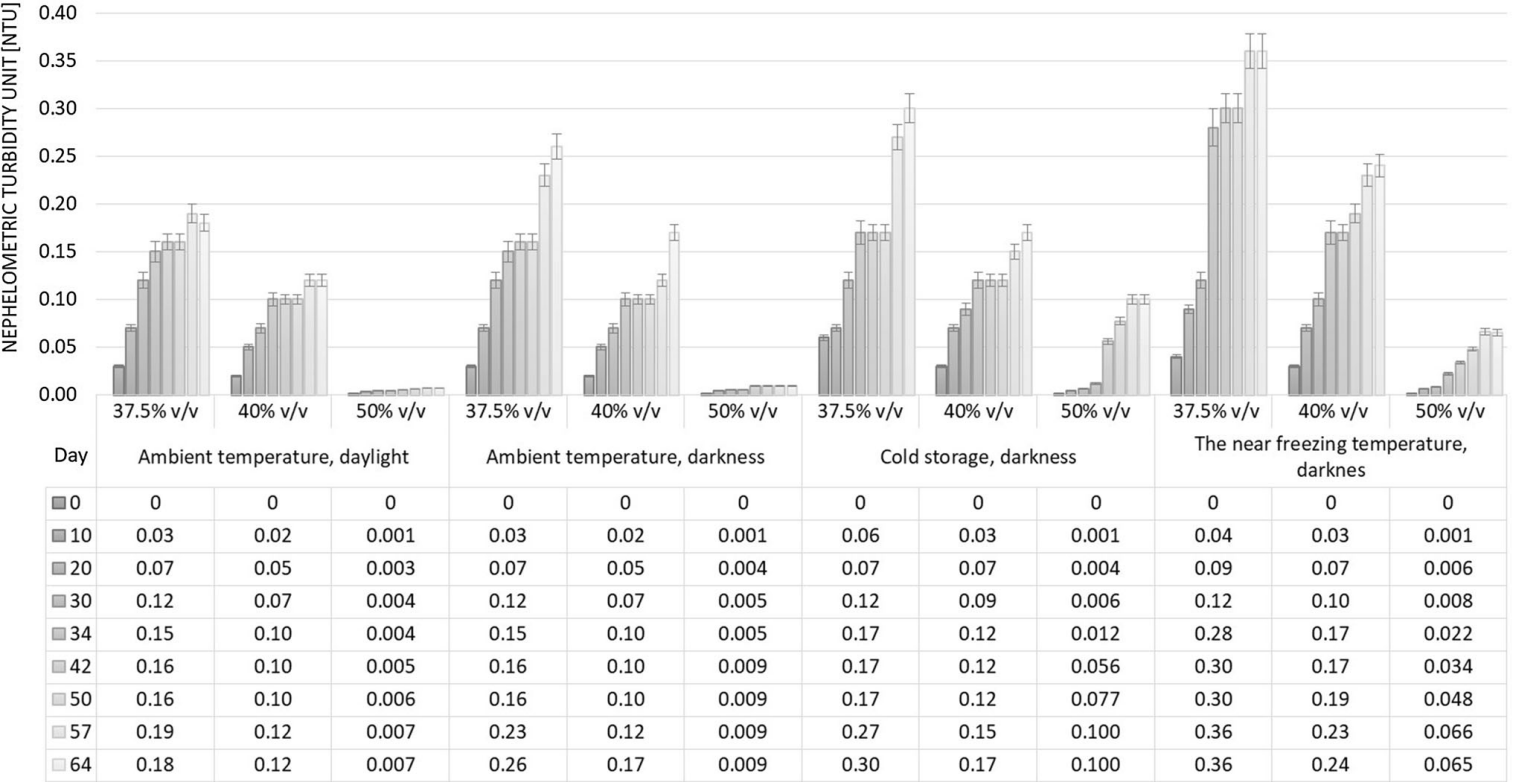
Effect of alcoholic strength by volume (ASV) and storage conditions on turbidity changes in solutions prepared from plum distillate with initial ASV of 81.92% v/v

The effects of ASV and storage conditions on the turbidity of the plum distillates were investigated. Both distillates were diluted from their initial ASVs of 76.77% v/v and 81.92% v/v to 37.5% v/v, 40% v/v and 50% v/v alcohol content, then stored for approx. 2 months under different conditions. The samples prepared from each of the plum distillates with an ASV of 50% v/v stored at ambient temperature (20 ± 2 °C) in daylight showed the least change, with no statistical difference in turbidity (*p* > 0.05) (Figs. 1, 2). A significant increase in turbidity was observed during the storage of samples with lower ASVs, i.e. 37.5% v/v and 40.0% v/v. Moreover, the samples from the distillate with an initial alcohol content of 76.77% v/v were characterized by significantly higher (*p* < 0.05) turbidity values than those prepared from spirit with an initial ASV of 81.92% v/v.

The samples with an alcohol content of 37.5% v/v prepared from distillate with a lower initial ASV (76.77% v/v) and stored at ambient temperature in daylight increased in turbidity from 1.29 NTU after 10 days to 6.40 NTU after 64 days (Fig. 1). Analogous samples prepared from plum distillate with an initial ASV of 81.92% v/v were much less hazy, with turbidity of only 0.18 NTU after 64 days (Fig. 2). Similar changes were observed in samples with 40% v/v alcohol content.

The second storage variant was ambient temperature (20 ± 2 °C) in darkness. Similar tendencies were observed to those described above for ambient temperature in daylight. However, the absolute values for turbidity were significantly higher (*p* < 0.05) than in the analogous samples stored in daylight. It may by supposed that, the UV rays in sunlight may act on the spirit solution, preventing the agglomeration of chemical compounds responsible for turbidity.

Cold storage (+ 8 °C) caused more intense turbidity, with higher NTU values in solutions with ASVs of 37.5% v/v and 40% v/v than in those with 50% v/v alcohol contents. Moreover, significantly higher values were observed in samples from plum distillate with an initial ASV of 76.77% v/v than in those prepared from spirit with 81.92% v/v alcohol content. This was probably due to the significantly higher (*p* < 0.05) content of volatile compounds in the distillate with a lower initial ASV.

Especially intense cloudiness was observed after only 10 days in samples with ASVs of 37.5% v/v and 40% v/v prepared from the distillate with an initial ASV of 76.77% v/v and stored at a temperature close to freezing (− 16 °C) (Fig. 1). After this period, the turbidity expressed in NTU reached values exceeding those determined after more than a month in samples subjected to cold storage (at + 8 °C). Further changes in the turbidity of the samples stored at near freezing temperature were not so dynamic. The samples with an ASV of 50% v/v stored at − 25 °C, especially those prepared from plum spirit with 81.92% v/v alcohol, showed relatively slight changes in turbidity, similar to those observed in analogous samples subjected to storage at + 8 °C (Figs. 1, 2).

The results summarized here show that storage temperature is a factor determining turbidity formation in plum distillates, especially in solutions with an ASV below 40% v/v. Lowering the temperature can accelerate the formation of turbidity, which can then be eliminated by filtration, thereby ensuring the physicochemical stability of the spirit.

### Effect of filtration through EUROPOR^®^ K40 filter sheets on concentrations of volatile compounds in plum distillates

Storage of the plum distillates was ended after 64 days, once no further changes in the turbidity of the samples were observed. All the samples were then filtered through EUROPOR^®^ K 40 filter sheets with a nominal filtration rate of 0.40–0.48 µm. Table 2 shows the changes in volatile compounds for samples produced from plum distillate with an initial ASV of 76.77% v/v. Similar results were obtained for samples from plum distillate with an initial ASV of 81.29% v/v (data not shown).

**Table 2:** Changes in the concentrations of volatile compounds in plum distillates (initial ASV 76.77% v/v) stored under different conditions and subjected to filtration trough a EUROPOR^®^ K 40 filter sheet

Compound [mg/L alcohol 100% v/v]	Control sample (before storage and filtration)	Ambient temperature (20 ± 2 °C), daylight			Ambient temperature (20 ± 2 °C), darkness		
		ASV [% v/v]					
		37.5	40	50	37.5	40	50
Alcohols							
1-propanol	515.30^c^ ± 43.20	432.36^b^ ± 39.26	427.55^b^ ± 41.36^c^	442.15^bc^ ± 40.22	439.22^bc^ ± 35.33	440.55^bc^ ± 39.62	433.52^bc^ ± 39.25
2-methyl-1-propanol	95.22^f^ ± 8.92	72.16^de^ ± 5.26	78.64^e^ ± 6.33	75.12^de^ ± 6.42	71.36^cde^ ± 5.45	73.31^de^ ± 5.72	72.21^de^ ± 6.26
1-butanol	6.90^h^ ± 0.54	3.11^bcdefg^ ± 0.25	3.52 ^g^ ± 0.28	3.35^efg^ ± 0.27	3.27^cdefg^ ± 0.25	3.35^efg^ ± 0.22^d^	3.42^fg^ ± 0.26
2-methyl-1-butanol	704.22^f^ ± 24.65	512.96^cd^ ± 15.56	574.83^e^ ± 17.32	530.35^d^ ± 18.55	533.10^d^ ± 16.89	530.27^d^ ± 19.38	530.78^d^ ± 20.35
3-methyl-1-butanol	2977.95^c^ ± 134.83	2520.85^b^ ± 72.35	2576.4^b^ ± 68.42	2630.42^b^ ± 75.15	2524.80^b^ ± 75.22	2526.66^b^ ± 69.66	2565.32^b^ ± 77.15
2-phenylethanol	2.00^c^ ± 0.18	1.65^b^ ± 0.13	1.72^bc^ ± 0.11	1.69^b^ ± 0.12	1.59^ab^ ± 0.13	1.62^b^ ± 0.12	1.57^ab^ ± 0.11
Methanol	49.65^a^ ± 6.76	49.96^a^ ± 5.39	47.86^a^ ± 6.22	49.96^a^ ± 5.39	45.73^a^ ± 6.36	47.86^a^ ± 5.78	46.2^a^ ± 6.15
Esters							
Ethyl acetate	193.23^c^ ± 15.94	163.65^b^ ± 7.56	148.76^a^ ± 6.78	155.65^ab^ ± 7.21	149.18^ab^ ± 6.89	149.63^ab^ ± 6.72	152.39^ab^ ± 6.63
2-methylbutyl acetate	3.36^b^ ± 0.25	2.86^a^ ± 0.21	3.15^ab^ ± 0.26	3.05^ab^ ± 0.28	2.74^a^ ± 0.26	2.87^ab^ ± 0.33	3.15^ab^ ± 0.24
Isoamyl acetate	7.58^a^ ± 0.91	7.06^a^ ± 0.12	6.74^a^ ± 0.08	6.65^a^ ± 0.09	6.93^a^ ± 0.15	6.75^a^ ± 0.09	6.88^a^ ± 0.12
2-phenylethyl acetate	33.15^a^ ± 4.26	33.56^a^ ± 3.15	35.78^a^ ± 2.69	34.77^a^ ± 3.33	33.16^a^ ± 3.34	34.55^a^ ± 3.22	34.16^a^ ± 3.17
Ethyl propanoate	0.78^a^ ± 0.09	0.68^a^ ± 0.08	0.71^a^ ± 0.09	0.73^a^ ± 0.07	0.76^a^ ± 0.06	0.77^a^ ± 0.06	0.68^a^ ± 0.06
Ethyl butanoate	2.89^a^ ± 0.33	2.55^a^ ± 0.30	2.69^a^ ± 0.41	2.57^a^ ± 0.36	2.49^a^ ± 0.36	2.53^a^ ± 0.35	2.60^a^ ± 0.37
Ethyl benzoate	0.39^b^ ± 0.03	0.27^a^ ± 0.02	0.30^a^ ± 0.02	0.28^a^ ± 0.02	0.27^a^ ± 0.02	0.29^a^ ± 0.02	0.30^a^ ± 0.02
Ethyl hexanoate	2.85^f^ ± 0.30	2.28^e^ ± 0.03	2.22^de^ ± 0.03	2.25^e^ ± 0.03	2.18^cd^ ± 0.03	2.15^bc^ ± 0.03	2.16^cd^ ± 0.03
Ethyl octanoate	10.29^c^ ± 0.18	10.12^c^ ± 0.11	10.03^c^ ± 0.11	10.03^c^ ± 0.13	10.27^c^ ± 0.22	10.39^c^ ± 0.20	10.35^c^ ± 0.22
2-methylpropyl octanoate	1.43^a^ ± 0.15	1.45^a^ ± 0.12	1.55^a^ ± 0.13	1.46^a^ ± 0.12	1.55^a^ ± 0.15	1.57^a^ ± 0.15	1.55^a^ ± 0.15
3-methylpropyl octanoate	1.60^a^ ± 0.05	1.52^a^ ± 0.15	1.55^a^ ± 0.15	1.55^a^ ± 0.13	1.57^a^ ± 0.16	1.60^a^ ± 0.13	1.54^a^ ± 0.15
Octyl octanoate	0.53^a^ ± 0.07	0.42^a^ ± 0.08	0.47^a^ ± 0.09	0.43^a^ ± 0.07	0.50^a^ ± 0.06	0.48^a^ ± 0.08	0.43^a^ ± 0.09
Decyl octanoate	0.22^a^ ± 0.04	0.23^a^ ± 0.05	0.20^a^ ± 0.04	0.23^a^ ± 0.05	0.22^a^ ± 0.04	0.20^a^ ± 0.05	0.24^a^ ± 0.06
Ethyl nonanoate	0.45^ab^ ± 0.02	0.43^ab^ ± 0.02	0.40^a^ ± 0.02	0.40^a^ ± 0.02	0.38^a^ ± 0.02	0.41^ab^ ± 0.03	0.40^a^ ± 0.02
Ethyl decanoate	7.75^b^ ± 0.68	7.29^b^ ± 0.25	7.89^b^ ± 0.33	7.19^b^ ± 0.35	7.36^b^ ± 0.12	7.65^b^ ± 0.26	7.65^b^ ± 0.22
Isobutyl decanoate	0.58^f^ ± 0.08	0.52^def^ ± 0.06	0.53^def^ ± 0.07	0.50^def^ ± 0.06	0.50^def^ ± 0.05	0.54^ef^ ± 0.05	0.54^ef^ ± 0.05
Ethyl tetradecanoate	1.17^f^ ± 0.21	0.72^c^ ± 0.03	0.71^c^ ± 0.03	0.86^e^ ± 0.03	0.76^cd^ ± 0.03	0.74^cd^ ± 0.02	0.79^cd^ ± 0.03
Carbonyl compounds and acetals							
Acetaldehyde	91.99^a^ ± 7.44	86.39^a^ ± 5.89	91.36^a^ ± 7.69	91.15^a^ ± 7.45	76.60^a^ ± 7.36	87.26^a^ ± 7.58	88.22^a^ ± 8.63
Isobutyr-aldehyde	2.24^c^ ± 0.29	1.96^c^ ± 0.22	2.09^c^ ± 0.15	2.19^c^ ± 0.15	1.94^c^ ± 0.15	2.21^c^ ± 0.26	2.15^c^ ± 0.17
Isovaler-aldehyde	0.52^bc^ ± 0.08	0.42^ab^ ± 0.02	0.46^bc^ ± 0.02	0.49^c^ ± 0.03	0.44^abc^ ± 0.02	0.47^c^ ± 0.02	0.48^c^ ± 0.03
2-methyl butyraldehyde	0.68^a^ ± 0.15	0.55^a^ ± 0.15	0.58^a^ ± 0.15	0.58^a^ ± 0.15	0.55^a^ ± 0.15	0.57^a^ ± 0.15	0.55^a^ ± 0.15
Benzaldehyde	0.83^a^ ± 0.17	0.80^a^ ± 0.12	0.81^a^ ± 0.15	0.79^a^ ± 0.13	0.81^a^ ± 0.12	0.79^a^ ± 0.09	0.77^a^ ± 0.11
2,3-butanodione	0.52^b^ ± 0.09	0.45^b^ ± 0.05	0.49^b^ ± 0.08	0.46^b^ ± 0.08	0.32^a^ ± 0.06	0.44^b^ ± 0.05	0.40^a^ ± 0.07
Acetaldehyde diethyl acetal	155.82^b^ ± 12.43	117.30^a^ ± 11.22	113.09^a^ ± 12.36	115.22^a^ ± 12.45	110.94^a^ ± 12.36	109.78^a^ ± 10.15	115.78^a^ ± 12.34

Compound [mg/L alcohol 100% v/v]	Cold storage (+ 8 °C), darkness			Near freezing temperature (from − 16 to − 25 °C), darkness		
	ASV [% v/v]					
	37.5	40	50	37.5	40	50
Alcohols						
1-propanol	352.36^a^ ± 30.22	327.55^a^ ± 31.34	315.78^a^ ± 33.15	332.36^a^ ± 39.22	327.55^a^ ± 41.36	330.66^a^ ± 44.15
2-methyl-1-propanol	61.92^abc^ ± 6.55	65.35^bcd^ ± 6.25	62.41^abcd^ ± 6.33	55.58^ab^ ± 6.33	50.19^a^ ± 6.45	53.75^ab^ ± 6.45
1-butanol	2.75^abc^ ± 0.28	2.77^abcd^ ± 0.32	2.66^ab^ ± 0.25	2.97^abcdefg^ ± 0.36	2.43^a^ ± 0.25	2.52^a^ ± 0.26
2-methyl-1-butanol	460.26^b^ ± 17.35	473.96^b^ ± 19.33	481.32^bc^ ± 19.22	416.94^a^ ± 18.35	423.92^a^ ± 19.56	443.96^ab^ ± 22.33
3-methyl-1-butanol	2212.24^a^ ± 68.32	2263.68^a^ ± 65.45	2279.62^a^ ± 63.49	2213.26^a^ ± 63.89	2246.5^a^ ± 65.33	2272.53^a^ ± 69.15
2-phenylethanol	1.32^a^ ± 0.14	1.34^a^ ± 0.11	1.32^a^ ± 0.16	1.34^a^ ± 0.11	1.42^a^ ± 0.12	1.39^a^ ± 0.11
Methanol	50.66^a^ ± 5.96	49.46^a^ ± 5.77	48.97^a^ ± 6.66	50.81^a^ ± 6.18	47.86^a^ ± 5.78	49.82^a^ ± 5.66
Esters						
Ethyl acetate	155.92^ab^ ± 7.15	150.76^ab^ ± 6.33	151.65^ab^ ± 7.21	155.23^ab^ ± 6.75	159.47^ab^ ± 6.89	149.76^a^ ± 7.12
2-methylbutyl acetate	2.88^ab^ ± 0.27	2.67^a^ ± 0.32	2.82^ab^ ± 0.29	2.69^a^ ± 0.33	2.82^ab^ ± 0.33	2.86^ab^ ± 0.27
Isoamyl acetate	6.61^a^ ± 0.15	6.65^a^ ± 0.07	6.72^a^ ± 0.08	6.59^a^ ± 0.08	6.73^a^ ± 0.08	6.68^a^ ± 0.07
2-phenylethyl acetate	30.56^a^ ± 3.24	31.52^a^ ± 3.17	30.66^a^ ± 2.85	30.78^a^ ± 3.22	31.46^a^ ± 3.15	30.55^a^ ± 3.36
Ethyl propanoate	0.71^a^ ± 0.07	0.73^a^ ± 0.08	0.70^a^ ± 0.06	0.66^a^ ± 0.05	0.68^a^ ± 0.06	0.68^a^ ± 0.09
Ethyl butanoate	2.49^a^ ± 0.38	2.55^a^ ± 0.37	2.49^a^ ± 0.37	2.49^a^ ± 0.36	2.53^a^ ± 0.41	2.48^a^ ± 0.39
Ethyl benzoate	0.28^a^ ± 0.02	0.29^a^ ± 0.02	0.29^a^ ± 0.02	0.26^a^ ± 0.02	0.29^a^ ± 0.02	0.29^a^ ± 0.02
Ethyl hexanoate	2.08^a^ ± 0.02	2.12^abc^ ± 0.03	2.10^ab^ ± 0.02	2.13^abc^ ± 0.03	2.08^a^ ± 0.02	2.08^a^ ± 0.02
Ethyl octanoate	8.51^b^ ± 0.15	8.63^b^ ± 0.09	8.52^b^ ± 0.15	8.17^a^ ± 0.16	8.15^a^ ± 0.12	8.13^a^ ± 0.17
2-methylpropyl octanoate	1.55^a^ ± 0.15	1.54^a^ ± 0.15	1.55^a^ ± 0.15	1.52^a^ ± 0.15	1.51^a^ ± 0.15	1.51^a^ ± 0.15
3-methylpropyl octanoate	1.59^a^ ± 0.13	1.61^a^ ± 0.15	1.55^a^ ± 0.14	1.45^a^ ± 0.12	1.44^a^ ± 0.13	1.45^a^ ± 0.12
Octyl octanoate	n.d.	n.d.	n.d.	n.d.	n.d.	n.d.
Decyl octanoate	n.d.	n.d.	n.d.	n.d.	n.d.	n.d.
Ethyl nonanoate	n.d.	n.d.	n.d.	n.d.	n.d.	n.d.
Ethyl decanoate	6.28^a^ ± 0.05	6.32^a^ ± 0.03	6.32^a^ ± 0.06	6.26^a^ ± 0.04	6.25^a^ ± 0.09	6.22^a^ ± 0.06
Isobutyl decanoate	0.34^bc^ ± 0.05	0.42 ^cd^ ± 0.05	0.44^cde^ ± 0.05	0.24^a^ ± 0.02	0.28^ab^ ± 0.04	0.24^a^ ± 0.02
Ethyl tetradecanoate	0.18^b^ ± 0.03	0.23^b^ ± 0.03	0.21^b^ ± 0.03	0.05^a^ ± 0.01	0.10^a^ ± 0.02	0.08^a^ ± 0.02
Carbonyl compounds and acetals						
Acetaldehyde	83.41^a^ ± 8.23	90.80^a^ ± 8.15	86.39^a^ ± 7.85	80.00^a^ ± 8.15	83.62^a^ ± 7.78	81.55^a^ ± 7.72
Isobutyr-aldehyde	1.61^b^ ± 0.15	2.31^c^ ± 0.21	2.11^c^ ± 0.25	1.22^a^ ± 0.15	1.43^ab^ ± 0.18	1.29^a^ ± 0.17
Isovaler-aldehyde	0.41^ab^ ± 0.05	0.42^ab^ ± 0.02	0.41^a^ ± 0.02	0.39^a^ ± 0.03	0.42^ab^ ± 0.02	0.40^a^ ± 0.02
2-methyl butyraldehyde	0.52^a^ ± 0.05	0.52^a^ ± 0.15	0.61^a^ ± 0.15	0.57^a^ ± 0.05	0.58^a^ ± 0.15	0.58^a^ ± 0.15
Benzaldehyde	0.77^a^ ± 0.11	0.82^a^ ± 0.14	0.79^a^ ± 0.11	0.69^a^ ± 0.08	0.77^a^ ± 0.11	0.75^a^ ± 0.09
2,3-butanodione	0.42^b^ ± 0.06	0.47^b^ ± 0.08	0.40^ab^ ± 0.06	0.31^a^ ± 0.05	0.48^b^ ± 0.06	0.42^b^ ± 0.05
Acetaldehyde diethyl acetal	122.05^a^ ± 10.69	121.74^a^ ± 11.78	120.74^a^ ± 12.98	125.16^a^ ± 12.15	125.33^a^ ± 12.36	121.54^a^ ± 12.75

*n.d* not detected; Mean values in rows with different superscript lowercase letters are significantly different (*p* < 0.05), as analyzed by the Tukey’s post hoc test

From a quantitative point of view, the most important volatile compounds in fruit spirits are higher alcohols, also known as fusel alcohols (Balcerek et al. 2017a, b). The principal constituents of the higher alcohols in the tested plum distillate samples were 3-methyl-1-butanol (isoamyl alcohol), 2-methyl-1-butanol (active amyl alcohol), 2-methyl-1-propanol (isobutanol) and n-propanol (1-propanol). This was in agreement with reports in the literature (Berry and Slaughter 1995; Soufleros et al. 2004). The amounts by which these compounds reduced during filtration were related to the turbidity of the samples. The smallest decreases in the majority of fusel alcohols were observed in samples stored at ambient temperature, the turbidity of which was relatively low. Lowering the storage temperature caused a higher agglomeration of volatiles, including higher alcohols, and resulted in a greater (*p* < 0.05) reduction in their content after filtration. The alcohol content (between 37.5 and 50% v/v) was not observed to have any effect on the adsorption of higher alcohols by the filtration sheet.

Methanol showed a different distribution from that of the higher alcohols. While methanol does not directly affect the flavour of the distillate, it is subjected to restrictive controls owing to its high toxicity (Cabaroglu and Yilmaztekin 2011). According to Regulation (EC) no. 110 (2008), the concentration of methanol in plum brandies should not exceed 12 g/L alcohol 100% v/v (i.e., 4.8 g/L alcohol 40% v/v). The concentration of methanol in the samples before filtration was below the maximum stipulated. Moreover, filtration did not significantly change the methanol concentration, regardless of the alcoholic strength of the samples or the storage conditions (Table 2).

Long chain ethyl esters, such as ethyl dodecanoate, ethyl hexadecanoate and ethyl-9-hexadecanoate compounds, may cause flocks or haziness in spirit beverages (Miljić et al. 2013; Pirie et al. 2000). These compounds behave as surfactants. They contain long hydrophobic carbon chains that prevent them from mixing with water, and a hydrophilic group. Moreover, under non-mixing conditions these fatty esters can behave as micelles, which are spherical clumps of lipid molecules in which the hydrophobic carbon tail points towards the center, away from water. The contribution that long chain ethyl esters make to causing cloudiness makes reduction of these compounds beneficial in terms of ensuring the stability of spirits (Carrillo and Cristobal 2015).

The filtration process resulted in various changes to the concentrations of esters. Regardless of the storage conditions, the filtration of samples through EUROPOR^®^ K40 filter sheets caused a similar (*p* > 0.05) decrease in the concentrations of ethyl acetate, 2-methylbutyl acetate, isoamyl acetate, 2-phenylethyl acetate, ethyl propanoate, ethyl butanoate, ethyl benzoate, ethyl hexanoate and ethyl octanoate, of not more than 30% in relation to the control. The concentrations of 2-methylpropyl octanoate and 3-methylpropyl remained constant.

Cold storage (+ 8 °C), as well as storage at near freezing temperatures (i.e. − 16 °C for ASVs of 37.5% v/v and 40% v/v, and − 25 °C for the ASV of 50% v/v) caused the elimination of octyl octanoate, decyl octanoate, ethyl nonanoate, whereas the contents of ethyl decanoate and isobutyl decanoate decreased by approx. 20% and 58%, respectively. In the samples stored at ambient temperature, both under daylight and in darkness, the concentrations of these compounds did not change as a result of filtration.

The esters that contribute most to the formation of haze in spirits are long chain fatty acid esters (C12–C16) (Hsieh et al. 2014; Miljić et al. 2013). As the alcohol strength falls below 45% v/v, the solubility of these compounds diminishes, leading to turbidity. This process is intensified at lower temperatures (Cai et al. 2016; Hsieh et al. 2014). With regard to fatty acid esters, only the presence of ethyl tetradecanoate was detected in the tested plum distillate. Its lowest reduction, by approx. 33% on average, was observed in the case of filtered samples that had been stored at ambient temperature. Cold storage (at + 8 °C) and filtration were associated with a decrease in ethyl tetradecanoate content of approx. 82%, whereas freezing the samples at − 16 °C or at − 25 °C (depending on the ASV) before filtration resulted in a reduction in the content of this compound of over 90% in comparison to the control.

Carbonyl compounds, i.e. aldehydes and ketones, are present in agricultural distillates including fruit spirits as by-products of fermentation. These compounds are intermediates of the two-step decarboxylation of alpha-keto acids to alcohols, as well as of the synthesis and oxidation of alcohols. They are often observed to have a negative influence on the quality of spirits (Plutowska et al. 2010). In agricultural distillates, aldehydes with five or more carbon atoms (i.e., valeraldehyde and isovaleraldehyde, hexanal) are particularly undesirable. Even at low concentrations, they may diminish the quality of spirits (Pielech-Przybylska et al. 2016).

The flavour of stone fruit spirits is affected by benzaldehyde, an aroma compound originating from the enzymatic degradation of the amygdalin present in the stones of the fruits. This passes into the mash during fermentation and later into the distillate (Christoph and Bauer-Christoph 2007). Moreover, alcoholic beverages can include diketone, i.e., 2,3-butanedione (diacetyl) with a buttery aroma, as well as acetals, which form rapidly in distillates. The most prominent of the latter group is acetaldehyde diethyl acetal (1, 1-diethoxyethane), the highest levels of which are found among whiskies, in particular malt whisky (Nykänen and Nykänen 1991).

After filtration through EUROPOR^®^ K40 filter sheets, the majority of carbonyl compounds and acetals did not show statistically significant changes in comparison to the control, irrespective of the storage conditions. Only the concentrations of isobutyraldehyde and isovaleraldehyde in samples placed in cold storage (+ 8 °C) or stored at near freezing temperatures (− 16 °C and − 25 °C) were reduced by a statistically significantly margin (*p* < 0.05) (Table 2).

### Effect of filter sheet type on the concentration of volatile compounds and the sensory characteristics of plum distillates

This part of the study was performed using plum distillate with an initial ASV of 81.92% v/v. This distillate was diluted to alcohol contents of 37.5, 40 and 50% v/v, and the samples were placed in cold storage at + 8 °C for 64 days. Next, the samples were filtered through one of two filter sheets with different nominal filtration rates, i.e. 0.40–0.48 μm (EUROPOR^®^ K 40) or 0.80 μm (BECO^®^ SELECT A20), or through an activated carbon filter sheet (BECO^®^ ACF 07). The results are presented in Table 3.

**Table 3:** Effect of filtration sheet type on changes to the concentrations of volatile compounds in plum distillates (initial ASV 81.92% v/v, cold storage at + 8 °C)

Compound [mg/L alcohol 100% v/v]	Control sample (before storage and filtration)	EUROPOR^®^ K 40 filter sheet		
		ASV [% v/v]		
		37.5	40	50
Alcohols				
1-propanol	395.52^c^ ± 29.50	215.78^b^ ± 23.15	227.55^b^ ± 21.34	252.36^b^ ± 22.22
2-methyl-1-propanol	86.40^d^ ± 6.05	61.92^c^ ± 6.55	65.35^c^ ± 6.25	62.41^c^ ± 6.33
1-butanol	1.65^g^ ± 0.23	0.59^c^ ± 0.04	0.66 ^cd^ ± 0.04	0.88^ef^ ± 0.06
2-methyl-1-butanol	696.40^d^ ± 36.05	467.69^c^ ± 31.05	497.38^c^ ± 30.15	495.33^c^ ± 30.05
3-methyl-1-butanol	2858.04^c^ ± 160.29	1283.75^a^ ± 110.33	1332.15^a^ ± 115.22	1355.28^a^ ± 118.19
2-phenylethanol	0.80^f^ ± 0.10	n.d.	0.22^a^ ± 0.03	0.32^b^ ± 0.02
Metanol	23.07^a^ ± 2.87	23.01^a^ ± 2.87	22.76^a^ ± 2.87	23.05^a^ ± 2.87
Esters				
Ethyl acetate	208.86^d^ ± 11.96	148.91^c^ ± 6.33	152.27^c^ ± 6.33	150.76^c^ ± 6.33
2-methylbutyl acetate	1.93^e^ ± .06	1.67^d^ ± 0.05	1.58^d^ ± 0.07	1.52^cd^ ± 0.15
Isoamyl acetate	5.26^b^ ± 0.84	4.35^b^ ± 0.12	4.65^b^ ± 0.07	4.57^b^ ± 0.12
2-phenylethyl acetate	20.72^b^ ± 2.15	18.52^b^ ± 1.24	19.12^b^ ± 1.17	19.06^b^ ± 2.05
Ethyl propanoate	0.56^b^ ± 0.06	0.49^b^ ± 0.05	0.50^b^ ± 0.06	0.53^b^ ± 0.05
Ethyl butanoate	1.72^b^ ± 0.15	1.59^b^ ± 0.08	1.65^b^ ± 0.07	1.74^b^ ± 0.07
Ethyl benzoate	0.19^d^ ± 0.03	n.d.	n.d.	0.17^cd^ ± 0.03
Ethyl hexanoate	2.51^e^ ± 0.39	2.08^d^ ± 0.02	2.12^de^ ± 0.03	2.10^de^ ± 0.02
Ethyl octanoate	8.47^ab^ ± 0.95	7.15^a^ ± 0.95	7.47^ab^ ± 0.95	7.47^ab^ ± 0.95
2-methylpropyl octanoate	0.78^e^ ± 0.09	0.35^bc^ ± 0.05	0.34^bc^ ± 0.05	0.35^bc^ ± 0.05
3-methylpropyl octanoate	1.23^c^ ± 0.14	1.19^c^ ± 0.13	1.11^c^ ± 0.15	1.15^c^ ± 0.14
Octyl octanoate	0.36^b^ ± 0.06	n.d.	n.d.	n.d.
Decyl octanoate	0.18^b^ ± 0.02	n.d.	n.d.	n.d.
Ethyl nonanoate	0.36^c^ ± 0.05	n.d.	n.d.	n.d.
Ethyl decanoate	0.36^b^ ± 0.06	n.d.	n.d.	n.d.
Isobutyl decanoate	0.32^b^ ± 0.08	n.d.	n.d.	n.d.
Ethyl tetradecanoate	0.13^c^ ± 0.05	0.03^a^ ± 0.01	0.05^b^ ± 0.01	0.05^b^ ± 0.01
Carbonyl compounds and acetals				
Acetaldehyde	92.28^a^ ± 7.04	85.34^a^ ± 7.22	87.26^a^ ± 7.15	88.56^a^ ± 8.14
Isobutyr-aldehyde	2.03^b^ ± 0.27	1.93^b^ ± 0.22	2.06^b^ ± 0.25	2.05^b^ ± 0.17
Isovaler-aldehyde	0.53^e^ ± 0.07	0.44^de^ ± 0.02	0.47^e^ ± 0.02	0.43^de^ ± 0.03
2-methyl butyraldehyde	0.78^b^ ± 0.11	0.72^b^ ± 0.08	0.72^b^ ± 0.09	0.69^b^ ± 0.06
Benzaldehyde	0.52^a^ ± 0.09	0.52^a^ ± 0.09	0.52^a^ ± 0.09	0.52^a^ ± 0.03
2,3-butanodione	0.35^a^ ± 0.09	0.33^a^ ± 0.05	0.39^a^ ± 0.06	0.36^a^ ± 0.09
Acetaldehyde diethyl acetal	145.78c ± 15.68	117.25a ± 11.85	121.08bc ± 12.68	120.79bc ± 12.05

Compound [mg/L alcohol 100% v/v]	BECO^®^ SELECT A 20 filter sheet			BECO^®^ ACF 07 activated carbon filter sheet		
	ASV [% v/v]					
	37. 5	40	50	37.5	40	50
Alcohols						
1-propanol	205.78^b^ ± 25.18	215.12^b^ ± 25.22	232.33^b^ ± 22.29	105.33^a^ ± 12.36	119.35^a^ ± 15.63	123.35^a^ ± 15.55
2-methyl-1-propanol	69.51^c^ ± 7.23	65.57^c^ ± 6.88	69.57^c^ ± 8.45	32.30^a^ ± 4.15	38.30^a^ ± 4.63	49.30^b^ ± 5.15
1-butanol	0.57^c^ ± 0.06	0.72^de^ ± 0.06	0.79^ef^ ± 0.06	0.28^a^ ± 0.03	0.39^b^ ± 0.03	0.36^b^ ± 0.03
2-methyl-1-butanol	258.78^a^ ± 26.55	269.26^a^ ± 26.18	361.23^b^ ± 30.24	267.93^a^ ± 25.22	277.38^a^ ± 29.27	259.30^a^ ± 26.63
3-methyl-1-butanol	1356.89^a^ ± 160.29	1415.60^ab^ ± 105.34	1556.62^b^ ± 122.23	1283.7^5a^ ± 145.24	1232.15^a^ ± 139.45	1296.25^ab^ ± 147.36
2-phenylethanol	0.39^cd^ ± 0.02	0.45^de^ ± 0.06	0.49^e^ ± 0.04	0.32^bc^ ± 0.05	0.36^bcd^ ± 0.04	0.38^bcd^ ± 0.04
Metanol	23.71^a^ ± 2.87	23.03^a^ ± 2.87	22.87^a^ ± 2.87	24.56^a^ ± 2.87	23.93^a^ ± 2.87	22.55^a^ ± 2.87
Esters						
Ethyl acetate	124.61^b^ ± 6.33	134.48^b^ ± 6.33	148.33^c^ ± 6.33	38.91^a^ ± 6.33	37.27^a^ ± 6.33	49.50^a^ ± 6.33
2-methylbutyl acetate	1.22^c^ ± 0.17	1.64^d^ ± 0.15	1.59^d^ ± 0.15	0.39^a^ ± 0.05	0.75^b^ ± 0.08	0.79^b^ ± 0.06
Isoamyl acetate	4.27^b^ ± 0.46	4.55^b^ ± 0.35	4.69^b^ ± 0.29	n.d.	n.d.	0.29^a^ ± 0.04
2-phenylethyl acetate	17.33^b^ ± 1.82	17.15^b^ ± 1.59	17.85^b^ ± 1.88	n.d.	n.d.	5.49^a^ ± 0.88
Ethyl propanoate	0.48^b^ ± 0.05	0.47^b^ ± 0.06	0.45^b^ ± 0.05	n.d.	n.d.	0.15^a^ ± 0.03
Ethyl butanoate	1.39^ab^ ± 0.18	1.65^b^ ± 0.17	1.24^a^ ± 0.17	n.d.	n.d	n.d.
Ethyl benzoate	0.13^c^ ± 0.02	0.16^cd^ ± 0.03	0.15^cd^ ± 0.02	n.d.	0.09^b^ ± 0.01	0.05^a^ ± 0.02
Ethyl hexanoate	1.55^c^ ± 0.12	1.95^de^ ± 0.22	2.05^de^ ± 0.25	n.d.	0.15^a^ ± 0.02	0.30^b^ ± 0.02
Ethyl octanoate	8.06^ab^ ± 0.22	8.19^ab^ ± 0.22	8.42^b^ ± 0.22	n.d.	n.d.	n.d.
2-methylpropyl octanoate	0.57^d^ ± 0.09	0.65^de^ ± 0.11	0.63^de^ ± 0.08	0.12^a^ ± 0.02	0.26^b^ ± 0.03	0.31^bc^ ± 0.03
3-methylpropyl octanoate	0.98^c^ ± 0.14	1.03^c^ ± 0.11	1.15^c^ ± 0.15	n.d.	0.11^a^ ± 0.03	0.19^b^ ± 0.04
Octyl octanoate	0.25^a^ ± 0.04	0.29^ab^ ± 0.06	0.29^ab^ ± 0.06	n.d.	n.d.	n.d.
Decyl octanoate	0.12^a^ ± 0.02	0.11^a^ ± 0.02	0.09^a^ ± 0.02	n.d.	n.d.	n.d.
Ethyl nonanoate	0.18^a^ ± 0.02	0.23^b^ ± 0.02	0.23^b^ ± 0.02	n.d.	n.d.	n.d.
Ethyl decanoate	0.18^a^ ± 0.06	0.22^a^ ± 0.06	0.25^ab^ ± 0.06	n.d.	n.d.	n.d.
Isobutyl decanoate	0.20^ab^ ± 0.03	0.22^ab^ ± 0.03	0.19^a^ ± 0.03	n.d.	n.d.	n.d.
Ethyl tetradecanoate	0.02^a^ ± 0.01	0.04^ab^ ± 0.01	0.05^b^ ± 0.01	n.d.	n.d.	0.03^a^ ± 0.01
Carbonyl compounds and acetals						
Acetaldehyde	83.37^a^ ± 9.04	85.62^a^ ± 8.54	87.15^a^ ± 7.89	85.32^a^ ± 7.14	87.26^a^ ± 7.34	86.22^a^ ± 8.04
Isobutyr-aldehyde	1.98^b^ ± 0.15	1.76^a^ ± 0.17	1.59^a^ ± 0.17	1.73^ab^ ± 0.22	1.78^ab^ ± 0.20	1.75^ab^ ± 0.22
Isovaler-aldehyde	0.41^cde^ ± 0.07	0.44^de^ ± 0.07	0.48^e^ ± 0.05	0.27^a^ ± 0.03	0.29^ab^ ± 0.03	0.35^bc^ ± 0.03
2-methyl butyraldehyde	0.53^a^ ± 0.06	0.55^a^ ± 0.07	0.49^a^ ± 0.05	0.52^a^ ± 0.04	0.52^a^ ± 0.07	0.60^a^ ± 0.05
Benzaldehyde	0.45^a^ ± 0.04	0.43^a^ ± 0.05	0.46^a^ ± 0.04	n.d.	n.d.	n.d.
2,3-butanodione	0.35^a^ ± 0.11	0.32^a^ ± 0.12	0.33^a^ ± 0.08	0.24^a^ ± 0.06	0.19^a^ ± 0.05	0.22^a^ ± 0.07
Acetaldehyde diethyl acetal	105.99ab ± 6.05	116.18b ± 7.22	109.45ab ± 8.26	97.02a ± 9.55	101.08ab ± 11.17	102.87ab ± 10.84

*n.d* not detected; Mean values in rows with different superscript lowercase letters are significantly different (*p* < 0.05), as analyzed by the Tukey’s post hoc test

When assessing the effect of filtration using different types of filter sheet on the reduction of volatiles in the plum distillates, it was observed that the BECO^®^ SELECT A20 filter sheet and the EUROPOR^®^ K 40 filter sheet were similarly effective (*p* > 0.05) at reducing the majority of higher alcohols. An advantage of using the BECO SELECT A20 filter sheet might be considered the lower reduction of 2-phenylethanol, which gives a pleasant aroma to distillates (Tešević et al. 2009).

As regards esters, there was a greater reduction in ethyl acetate and 2-methylbutyl acetate in samples filtered through BECO SELECT A20, in comparison to EUROPOR^®^ K 40. The concentrations of ethyl hexanoate, ethyl octanoate, 2-methylpropyl octanoate and 3-methylpropyl octanoate were similar in all the filtered samples, regardless of whether EUROPOR^®^ K 40 or BECO SELECT A20 filter sheets were used. Moreover, the BECO SELECT A20 filter sheet gave distillates that contained esters such as octyl octanoate, decyl octanoate, ethyl nonanoate, ethyl decanoate and isobutyl decanoate, which were eliminated when the EUROPOR^®^ K 40 filter sheet was used.

There were no statistically significant changes (*p* > 0.05) in the concentrations of aldehydes and acetals between the samples filtered through EUROPOR^®^ K 40 or BECO SELECT A20, compared to the control.

The highest reduction in the majority volatiles, with the exception of carbonyl compounds, was observed following filtration with the activated carbon filter sheet. The decrease in the concentrations of volatiles was not strictly correlated with the alcohol content, although lowering the alcohol content (from 50 to 37.5% v/v) did contribute to increased precipitation of volatile compounds as cloudiness/haze, which could be eliminated during filtration.

The filtration of plum distillate samples through a BECO^®^ ACF 07 activated carbon filter sheet resulted in a decrease in the content of higher alcohols of between approx. 41% (3-methylo-1-butanol) and approx. 83% (1-butanol) compared to the control. An especially large reduction was observed in the case of fatty acid esters. Short chain fatty acid esters are highly desirable from the perspective of aroma and taste. Medium and long-chain fatty acid esters are haze-inducing, and long-chain fatty acid esters have been shown to be a prime cause of off-odors and off-tastes (Miljić et al. 2013). Use of the activated carbon filter sheet resulted in high and non-selective reduction of fatty acid esters. The concentrations of acetate esters (i.e. ethyl acetate, 2-methylbutyl acetate, isoamyl acetate, 2-phenylethyl acetate) decreased by approx. 90%. Very large reductions (of more than 90%) were also observed in the case of esters C6–C10, as well as for C14 (Table 3).

A different tendency was observed in the case of carbonyl compounds. For instance, the concentration of acetaldehyde did not change in any of the filtered samples, in comparison to the control. The low adsorption capacity of acetaldehyde on the surface of activated carbon is associated with its relatively high vapor pressure (Yao 2008). Filtering the plum distillate samples through activated carbon-based filter sheet caused decreases in the concentrations of other aliphatic aldehydes, as well as of 2,3-butanedione and acetaldehyde diethyl acetal, of up to approx. 49% in comparison to the control. The only compound which was eliminated from the tested samples by filtration was benzaldehyde. Donor–acceptor complexes may form in compounds such as benzaldehyde, between the aromatic ring and the surface of the adsorbent. The presence of an electron-withdrawing carboxyl group on the aromatic ring may lower the electronic density of the molecules, making it easier for the aromatic ring to act as an acceptor. This could explain the higher absorbance of benzaldehyde (Mattson et al. 1969).

All the plum distillates after filtration were re-exposed to cold storage (+ 8 °C) for approx. 4 wks. No turbidity was observed in any of the samples. This confirms that cold storage followed by filtration is an effective way to ensure the clarity of plum brandies. However, given that filtration reduces the concentration of volatile compounds in plum distillates, it is important to choose the most appropriate filter sheet, to preserve the original flavour and aroma. The plum distillate samples were therefore submitted to sensory evaluation. It was found that sensorial differences existed between the filtered samples and the control plum distillate (before storage and filtration). These differences especially concerned odor and taste (Table 4).

**Table 4:** Sensory assessment of tested plum distillate samples

Sample	Sensory parameters				
	Color	Clearness	Odor	Taste	Overall acceptance
Control sample (before storage and filtration)	9.0 ± 0.0^a^	8.3 ± 0.2^a^	8.4 ± 0.2^c^	7.6 ± 0.3^b^	8.3 ± 0.2^b^
EUROPOR^®^ K40 filter sheet					
37.5% v/v	9.0 ± 0.0^a^	8.6 ± 0.3^ab^	7.5 ± 0.2^b^	7.6 ± 0.3^b^	8.2 ± 0.1^b^
40% v/v	9.0 ± 0.0^a^	8.9 ± 0.1^bc^	7.6 ± 0.2^b^	7.5 ± 0.1^b^	8.3 ± 0.1^b^
50% v/v	9.0 ± 0.0^a^	9.0 ± 0.0^c^	7.5 ± 0.2^b^	7.5 ± 0.1^b^	8.3 ± 0.2^b^
BECO^®^ SELECT A20 filter sheet					
37.5% v/v	9.0 ± 0.0^a^	9.0 ± 0.0^c^	8.7 ± 0.2^c^	8.5 ± 0.1^c^	8.8 ± 0.3^c^
40% v/v	9.0 ± 0.0^a^	9.0 ± 0.0^c^	8.6 ± 0.2^c^	8.4 ± 0.2^c^	8.8 ± 0.2^c^
50% v/v	9.0 ± 0.0^a^	9.0 ± 0.0^c^	8.8 ± 0.2^c^	8.5 ± 0.1^c^	8.8 ± 0.1^c^
BECO^®^ ACF 07 activated carbon filter sheets					
37.5% v/v	9.0 ± 0.0^a^	8.3 ± 0.3^a^	6.7 ± 0.2^a^	5.5 ± 0.1^a^	7.4 ± 0.2^a^
40% v/v	9.0 ± 0.0^a^	8.5 ± 0.2^a^	6.6 ± 0.2^a^	5.4 ± 0.2^a^	7.4 ± 0.2^a^
50% v/v	9.0 ± 0.0^a^	8.5 ± 0.2^a^	6.6 ± 0.2^a^	5.5 ± 0.1^a^	7.4 ± 0.2^a^

Mean values in columns with different superscript lowercase letters are significantly different (*p* < 0.05), as analyzed by the Tukey’s post hoc test

The color, clearness, odor, taste and overall acceptability of the control sample were placed by the panel of judges in the category ‘like moderately’. Filtration through EUROPOR^®^ K 40 sheets gave the brandies acceptable overall sensory characteristics, which judges also rated as ‘like moderately’, despite some differences that were noticed in odor and taste. In the case of samples filtered through the EUROPOR^®^ K 40 filter sheet, the three testers described a slight decline in organoleptic properties compared to the control. However, this was not reflected in the scores. The highest scores in the sensory evaluation of treated brandies were given to the samples filtered through the BECO^®^ SELECT A20 filter sheet. These samples were assessed as better and more harmonized in terms of aroma and flavor than the control sample. They were placed in the ‘like very much’ category. As a consequence, this filter sheet can be considered a suitable choice for practical applications. The BECO^®^ ACF 07 filter sheet gave the purest samples, with little of the specific character of the raw material, plums. This resulted in a significant decrease in sensory scores, especially with regard to odor and taste, which was reflected by the classification score “like slightly.”

## Conclusion

This study has shown that volatile compounds, including higher alcohols and fatty acids esters, are the major cause of the turbidity that appears during storage of plum brandies. The best solution for eliminating turbidity while improving the organoleptics of the tested plum distillates was found to be treatment consisting of cold storage at + 8 °C for approx. 2 month followed by filtration through a filter sheet with a nominal retention rate of 0.8 µm (BECO^®^ SELECT A20). It may be hypothesized that further lowering the temperature could shorten the time needed to obtain higher turbidity, which can then be removed in the filtration step. The activated carbon filter sheet BECO^®^ ACF 07 caused the largest decrease in the concentration of the majority of volatiles. As a consequence, the filtered distillate received the lowest scores in a sensory assessment. Cold storage and subsequent filtration through properly selected filter sheets can be considered an effective of ensuring both the stability (in terms of lack of turbidity) and sensory quality of plum brandies.
